# Bibliometric analysis of research trends and advancements in medicinal plant microbiome

**DOI:** 10.3389/fpls.2024.1495198

**Published:** 2024-11-20

**Authors:** Danling Hu, Lizhe Hu, Ouli Xiao, Jieyin Chen, Xiaofeng Dai, Yongwei Sun, Zhiqiang Kong

**Affiliations:** ^1^ State Key Laboratory for Biology of Plant Diseases and Insect Pests, Institute of Plant Protection, Chinese Academy of Agricultural Sciences, Beijing, China; ^2^ Key Laboratory of Forage and Endemic Crop Biotechnology, Ministry of Education, School of Life Sciences, Inner Mongolia University, Hohhot, China; ^3^ Western Agricultural Research Center, Chinese Academy of Agricultural Sciences, Changji, China

**Keywords:** CiteSpace, bibliometry, medicinal plants, microorganisms, nature product

## Abstract

Medicinal plants and microorganisms are closely linked, with microorganisms boosting plant growth, offering pest control, and enhancing secondary compound production. However, there’s a lack of systematic research, detailed molecular studies, and standardized methods for effectively using microorganisms in developing products from medicinal plants. To enhance understanding of the present research progress, emerging patterns, and key areas pertaining to microorganisms found in medicinal plants, CiteSpace bibliometric software was employed to visualize and analyze 1269 English publications sourced from the Science Net Core Collection database. Through the utilization of keyword co-occurrence analysis and cluster analysis methods, this study seeks to explore collaborative networks among countries, institutions, and scholars involved in the study of microorganisms in medicinal plants. This review highlights key research areas in microbiology, focusing on evaluating natural compounds for antibacterial properties and the impact of secondary metabolites on microbial communities, aiming to highlight significant research domains and primary focuses for researchers and professionals engaged in the field of microbiology concerning medicinal plants.

## 1 Introduction

Medicinal plants are botanical species containing bioactive constituents in various tissues such as roots, stems, leaves, flowers, and fruits, which provide therapeutic benefits for human health. These plants have been utilized for disease prevention, treatment, or mitigation (Gurib-Fakim, 2006). The ancestral knowledge regarding the therapeutic attributes of medicinal plants has been transmitted across different societies over time, establishing the basis for traditional medical customs that have been employed for thousands of years (Khan, 2014). Medicinal plants have unique pharmacological effects due to bioactive compounds like alkaloids and flavonoids, offering therapeutic benefits such as antibacterial, anti-inflammatory, pain relief, antioxidant, and immune modulation, essential for treating various diseases (Morsy, 2014; Ramawat et al., 2009). Scientific research has validated the medicinal benefits of many plant species, leading to their widespread clinical use (Pferschy-Wenzig and Bauer, 2015). Key medicinal plants such as *Panax ginseng*, *Angelica sinensis*, *Astragalus membranaceus* root, *Lycium barbarum*, and others are crucial in traditional Chinese medicine and modern pharmacology. With the growing interest in natural remedies, continued research and development of these medicinal plants will remain a focal point of attention.

Medicinal plants have a strong connection with microorganisms, encompassing various microbial communities such as bacteria, fungi, actinomycetes, and other organisms. These microorganisms primarily inhabit the rhizosphere of these plants, with endophytic bacteria engaging in plant-microbe interactions within the plant tissues (Alawiye and Babalola, 2019). The interactions between these microorganisms and medicinal plants are complex, microorganisms can indirectly influence the growth and therapeutic properties of medicinal plants by enhancing plant growth and increasing their resilience to stress (Kumar et al., 2022). Additionally, certain microorganisms can produce bioactive compounds themselves, with specific strains capable of synthesizing compounds similar to those in medicinal plants. Hence, the relationship between microorganisms and medicinal plants extends beyond a simple symbiotic alliance, evolving into a diverse ecosystem characterized by mutual progress and influence. Effective management of microbes in medicinal plant cultivation can enhance their yield and quality, ensuring effective and safe herbal treatments (Huang et al., 2018). Furthermore, exploring how microorganisms interact with medicinal plants helps discover new medicinal sources, modernize traditional Chinese medicine, and reuse traditional remedies (Wang et al., 2022).

Given the intensification of anthropogenic activities and continuous alterations in microbial communities, it is imperative to investigate recent research advancements and emerging trends concerning the microbiota associated with medicinal plants. By delving deeper into the intricate interactions between microorganisms and medicinal plants, we can attain a comprehensive understanding of their mutual relationships, thereby providing a more scientifically robust basis for the conservation and utilization of medicinal plant resources. Hence, relying on the core collection database of Web of Science™ (WoS), this study conducted a systematic analysis of the literature pertaining to “microorganisms of medicinal plants” by using bibliometric methods and visualization analysis based on CiteSpace software. The objective of this research is to comprehensively examine and elucidate the beneficial roles of microorganisms associated with medicinal plants in relation to the enhancement of plant growth, health, and their medicinal properties.

## 2 Data collection and methods

### 2.1 Data collection

**Figure 1:**
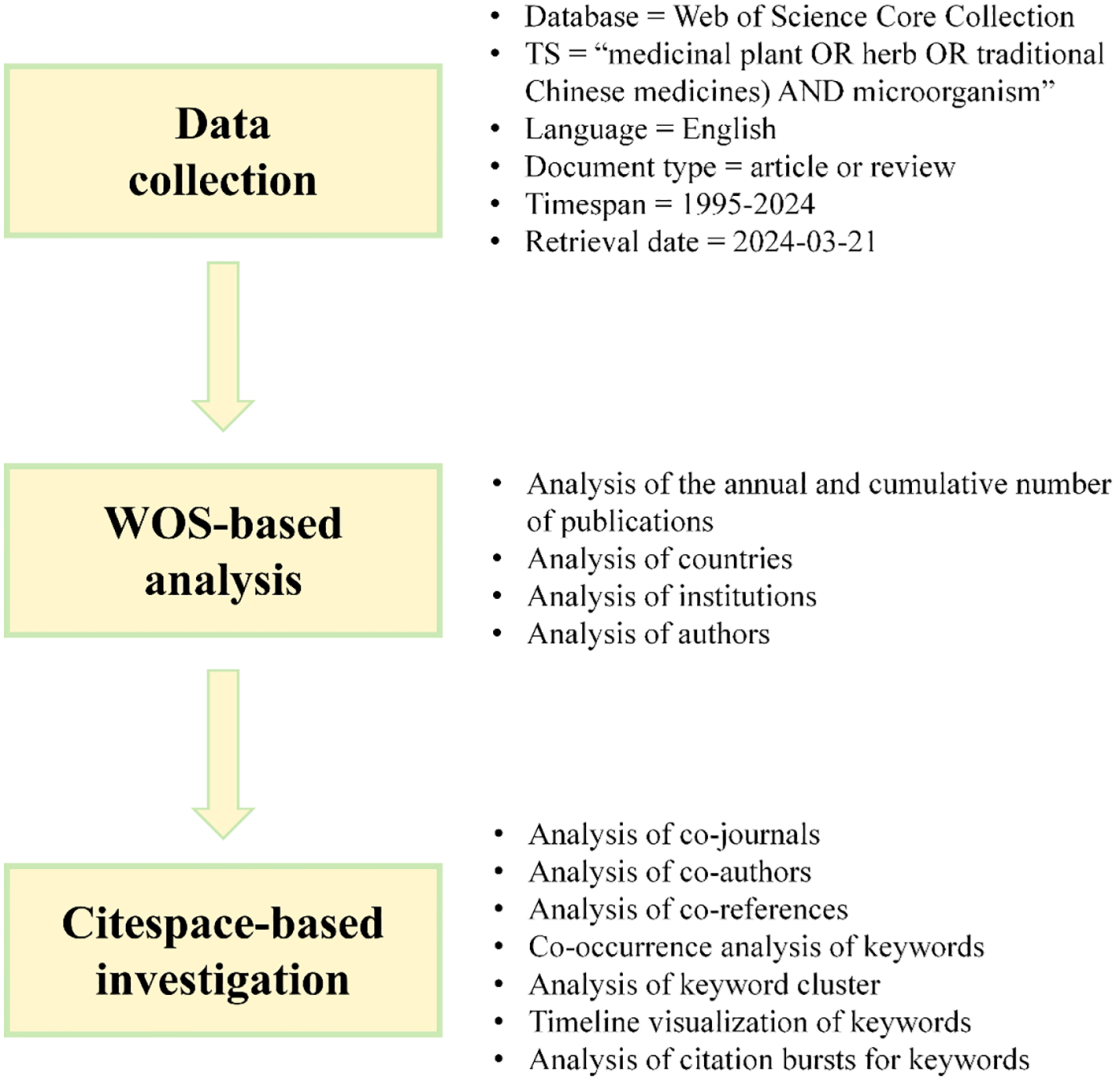
.The flowchart shows the methods used for bibliometric analysis.

This study utilizes the WoS core collection as a data-gathering platform, based on the data sources needed in CiteSpace (**Figure 1**), the retrieval formula of this subject was TS = (medicinal plant OR herb OR traditional Chinese medicines) AND microorganism. The search criteria specified were: language = English, document types = article or review, and a time span from 1995 to 2024. The retrieval date was March 21, 2024, and a total of 1297 English-language papers were obtained. After excluding studies not pertinent to the research topic, a final corpus of 1,269 papers was obtained. The literature chosen for analysis was obtained by downloading it in the form of “full records and cited references” and then saved as unformatted text files, serving as data samples.

### 2.2 Research method

With the swift progression of social network analysis and graphic visualization science, researchers increasingly utilize statistical metrological visualization software to perform extensive bibliometric analyses of scientific research knowledge (Chen et al., 2010). CiteSpace, a software tool, applies sophisticated mathematical and statistical methods for in-depth data mining of scientific literature. By visually depicting the structure, patterns, and relationships within the literature, it aids researchers in obtaining deep insights into the developments, focal areas, and emerging trends within a specific research domain (Chen, 2004, 2006). In this study, the CiteSpace version 6.1.R6 was utilized to convert and eliminate duplicate records in the literature acquired from the WoS core collection database.

The time slice was set to one year, and the node types were selected as author, institution, country, and keyword. Ultimately, a collaborative network diagram was constructed to illustrate the analysis of authorship, institutional affiliation, and geographical location, grounded in co-occurrence clusters.

## 3 Results and discussion

### 3.1 Publication trend analysis

**Figure 2:**
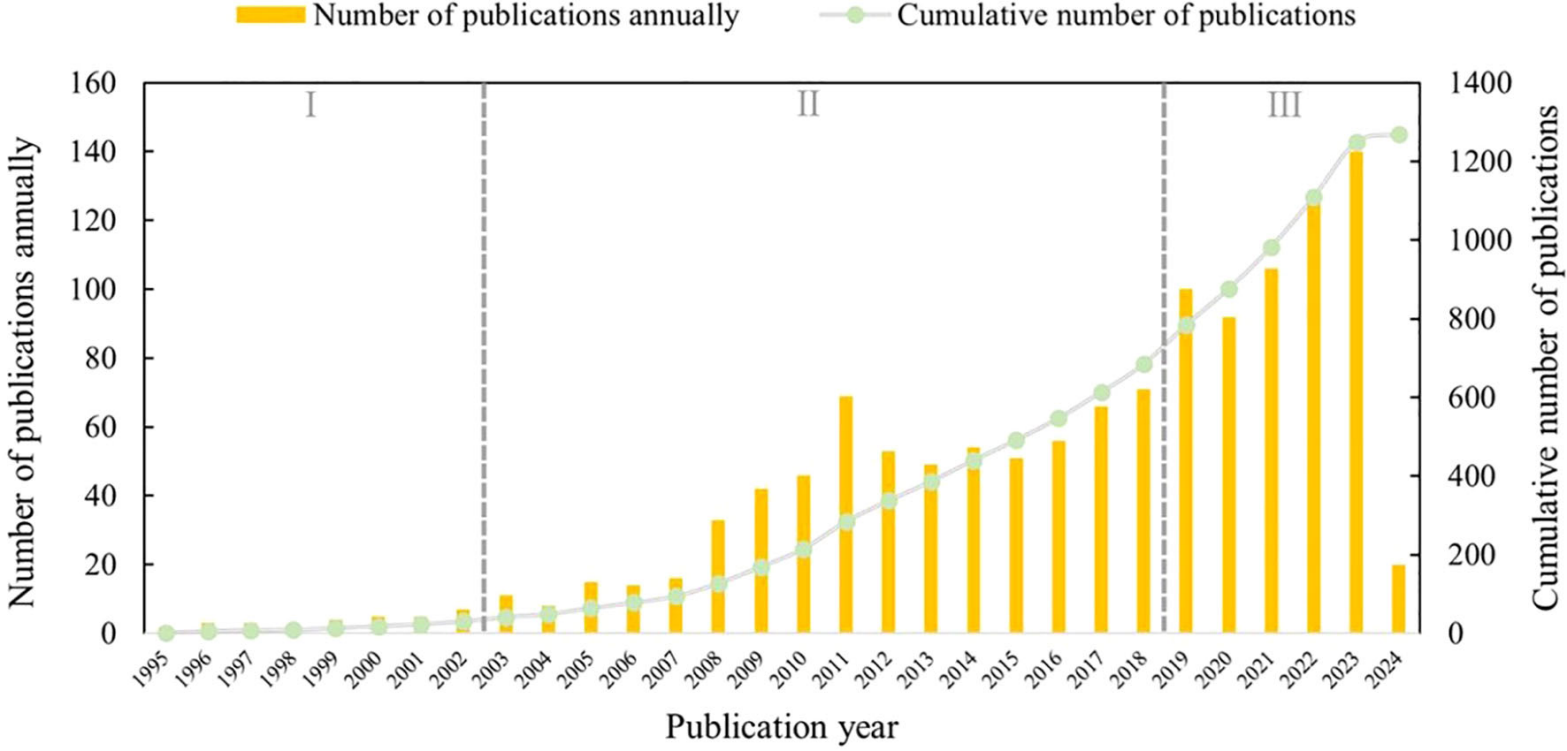
Trends in the annual and cumulative number of publications on microorganisms in medicinal plant research.

The data analysis results of the literature survey on microorganisms present in medicinal plants, obtained from the WoS database, are visually represented in **Figure 2**. In general, there has been a consistent increase in the number of publications focusing on microorganisms found in medicinal plants, which can be categorized into three distinct sections.

During the data collection period of this study (1995-2024), the earliest English-language publication concerning microbes associated with medicinal plants was authored by Izzo et al. (1995). Their comprehensive biological screening aimed to assess the antibacterial properties of Italian medicinal plants, revealing that 64 out of 68 extracts (94.1%) exhibited inhibitory effects against one or more microbial strains.

The initial phase of progress occurred between 1995 and 2002, a period during which the exploration of medicinal plant microorganisms was in its formative stage. Throughout these eight years, the annual number of publications remained below ten. During this time, scholars began to recognize the concept and significance of medicinal plant microorganisms, thereby initiating pertinent research endeavors. The subsequent phase spanned from 2003 to 2018, witnessing a steady increase in the annual publication count. This trend can be attributed to the rapid advancements in microbiome science. In stark contrast, the most recent quinquennium has experienced an exponential surge in publication numbers each year. This five-year period is particularly significant, as it accounts for 46% of the papers published over the course of three decades.

After understanding the characteristics and development stages of these published articles, analyzing collaborative networks can help identify key contributors and collaboration patterns, thereby facilitating interdisciplinary research and discovering collaboration hotspots.

### 3.2 Cooperation network analysis

Collaborative analysis facilitates a comprehensive understanding of the diverse nations, institutions, and authors that have significantly contributed to the field of medicinal plant microbiology through collaborative networks (Gazni et al., 2012).

#### 3.2.1 Introduction to country cooperation dynamics

**Figure 3:**
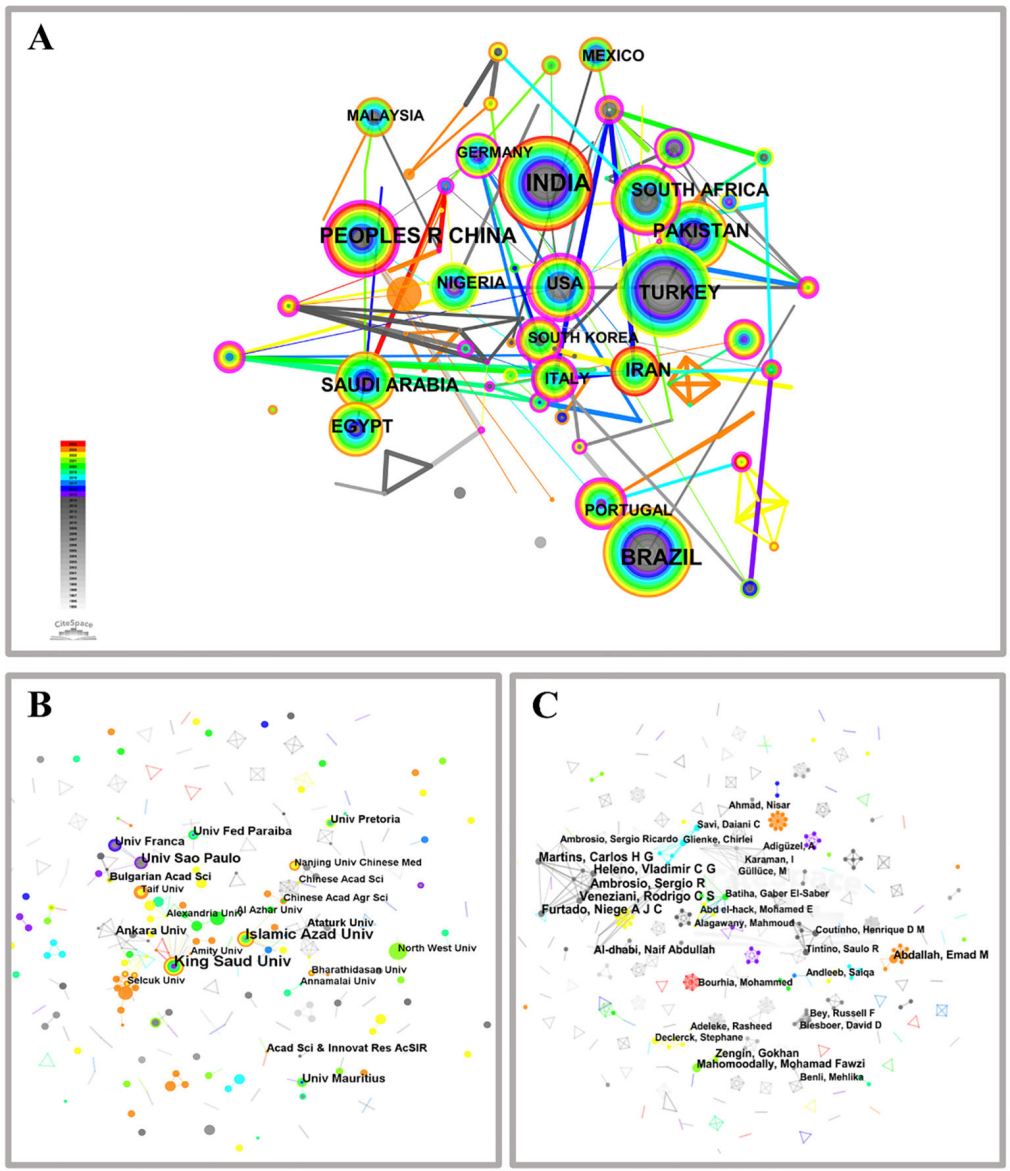
Collaborative network of country **(A)**, publishing institutions **(B)** and authors **(C)** on microorganisms in medicinal plant research.

The “country” parameter was selected in the CiteSpace analysis software, and **Figure 3A** illustrates the co-occurrence network of countries within the research domain of medicinal plant microorganisms. The nodes in this figure exhibit varying sizes, representing different countries that have published articles, distinguished by color and size. Larger nodes are indicative of a greater quantity of published papers, whereas smaller nodes correspond to a lesser number of publications. Additionally, these nodes are encompassed by purple rings of various sizes, thicker lines correspond to higher centrality levels (Osareh, 1996).

The collaboration network between countries (**Figure 3A**; N = 112, E = 148, Density = 0.0238) reveals a noteworthy extent of global cooperation among nations. India emerges as the leading contributor with the highest number of publications on the topic, totaling 230, followed by Brazil (151), China (111), Turkey (86), and Saudi Arabia (68) (**Supplementary Material Table S1**). However, despite its substantial publication count, India exhibits a centrality value of 0, indicating a lack of collaborative efforts with other countries. Among the top three countries in terms of publication volume, China demonstrates the most significant centrality. In recent years, China has fostered greater collaboration with Germany, Switzerland, Mongolia, Sri Lanka, and Tanzania in terms of node connections. Furthermore, the United States demonstrates the highest centrality (centrality = 0.42) among the top ten countries based on publication count and also possesses the greatest international influence within this field.

To summarize, countries with high centrality are instrumental in advancing research in the field of medicinal plant microbiology. Despite China’s significant investment of time and effort to enhance its publication output, it has not yet attained the centrality achieved by less prolific nations such as the United States, England, and Spain. The comparatively low centrality of China’s publications in this field can be ascribed to several factors, including linguistic diversity, the interdisciplinary nature of academic domains, and varying scholarly perspectives.

#### 3.2.2 Introduction to institutional cooperation dynamics

Cooperation network analysis was conducted on institutions that published articles on medicinal plant microorganisms (**Figure 3B**; N = 570, E = 395, Density = 0.0024). King Saud University emerged as the top-ranking institution in the entire cooperation network, having published a remarkable number of 20 articles and establishing close collaborations with various esteemed institutions such as the University of Nizwa, Damanhour University, KwaZulu-Natal University, and Suez Canal University. Conversely, other research institutions exhibited a more fragmented collaboration pattern in the field of medicinal plant microorganisms with a centrality score of zero. Notably, the gray lines depicted in **Figure 3B** indicate instances where certain institutions displayed loose cooperation but had made pioneering contributions to this domain.

The institutions with the highest publication output and center status, as shown in **Supplementary Material Table S2**, are listed below. King Saud University (20) claims the top spot, followed by Islamic Azad University (17), University of Sao Paulo (13), University of Mauritius (9), Ankara University (9), Federal University of Paraiba (9), Universidade de Franca (9), Ataturk University (7), Bulgarian Academy of Sciences (7), and University of Pretoria (7). With regards to institutional classification, all except one belongs to universities. Universities contribute significantly with a total publication count of 1106 articles, accounting for approximately 87% of the overall article output. The core technology of these plants primarily originates from universities with robust research capacity. In addition, the Chinese Academy of Sciences and Nanjing University of Chinese Medicine are well-known establishments in China due to their significant contributions in publishing research papers related to microbiology of medicinal plants. However, they rank 19th and 21st globally.

#### 3.2.3 Introduction to author cooperation dynamics

The results of the author collaboration network analysis reveal a significant dispersion among researchers in this field, as depicted in **Figure 3C**. Small team studies are prevalent, limited communication, and insufficient cross-regional cooperation. Notably, within the network diagram’s central region, the largest subnetwork consists of five nodes representing Furtado, Niege A J C; Martins, Carlos H G; Veneziani, Rodrigo C S; Ambrosio, Sergio R; and Heleno Vladimir C G. However, their research primarily focuses on investigations into medicinal plant microorganisms. Furthermore, **Supplementary Material Table S3** indicates that all these authors are affiliated with institutions in Brazil—highlighting Brazil’s pioneering contributions to this field. Other prolific authors include Mahomoodally Mohamad Fawzi (4) from Vietnam; Al-Dhabi Naif Abdullah (4) and Abdallah Emad M (4) from Saudi Arabia; Zengin Gokhan (4) from Turkey; and Adeleke Rasheed from South Africa. The individuals with the highest publication frequency in the previous one to two years are Abdallah, Emad M and Bourhia, Mohammed.

In summary, based on the number of published papers, the largest and most productive team members are affiliated with institutions in Brazil, while other authors tend to collaborate within their respective countries. This observation suggests that the author’s collaborative network in medicinal plant microbiology research has limited international connections and corresponding author communications. Therefore, efforts should be made to enhance international academic exchanges and foster friendly contacts and collaborations with scientific communities in Brazil and other countries. In CiteSpace, initially conducting a cooperation network analysis can reveal relationships among researchers, aiding in the identification of core contributors and collaboration patterns. Subsequently, a co-citation analysis explores interrelationships among literature to identify key works and research trends, thereby guiding future research directions.

### 3.3 Co-citation analysis

If two or more documents contain multiple instances of the same author, an association can be established between them. Co-Citation networks serve as a highly suitable scientific tool for assessing the scholarly impact of journals, literature, and authors, thereby assisting researchers and institutions in discovering valuable information (Small, 1973).

#### 3.3.1 Network construction: mapping journal co-citation relationships

**Figure 4:**
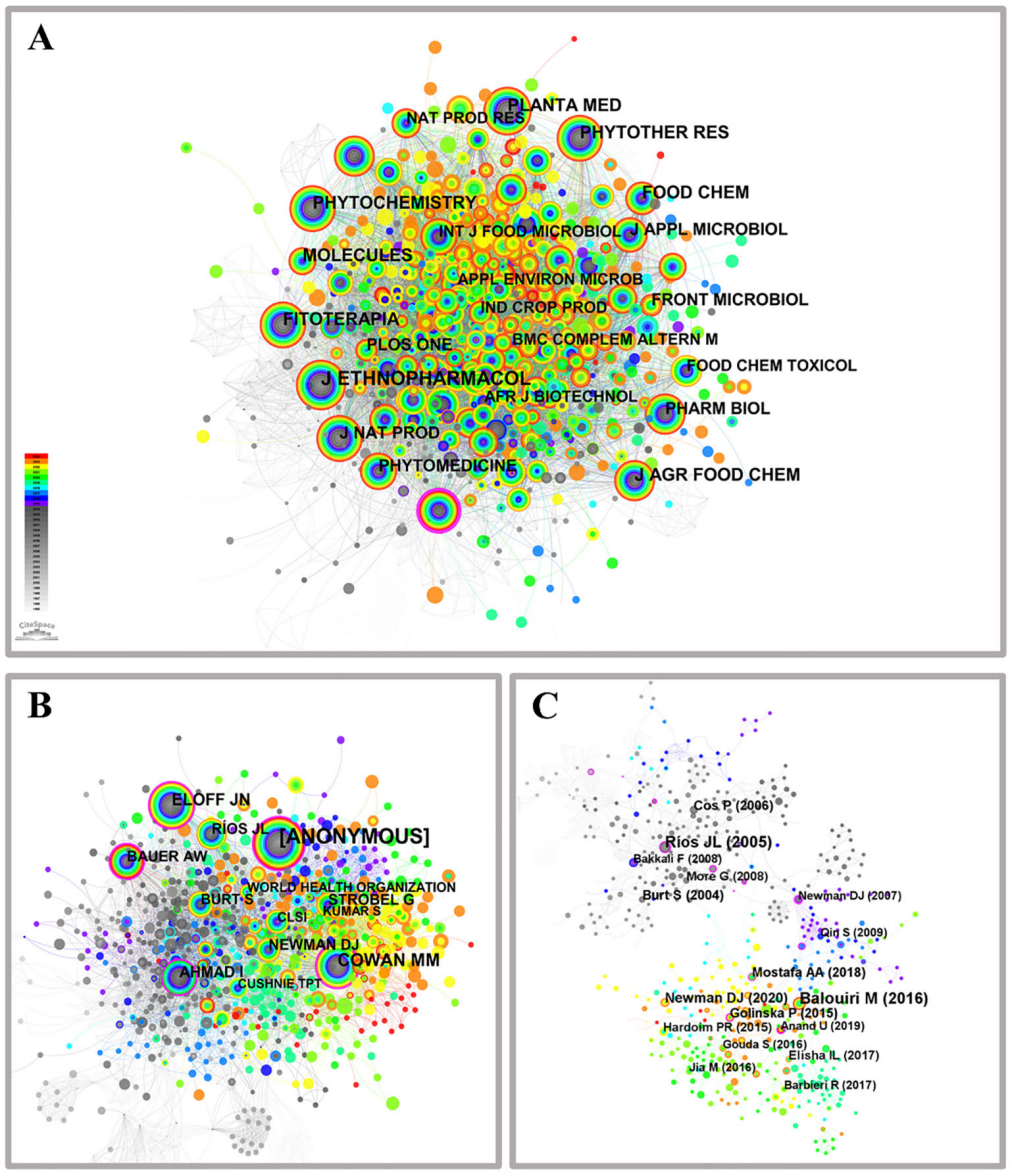
Co-citation network of journals **(A)**, authors **(B)** and references **(C)** on microorganisms in medicinal plant research.

The “cited journals” parameter in CiteSpace analysis software was used to identify and visualize the co-citation network of journals about medicinal plant microbiology research, as depicted in **Figure 4A**. The nodes within this network represent distinct journals that have been co-cited in relevant documents, distinguished by their color and size. Notably, larger nodes indicate a higher degree of scholarly attention received. **Supplementary Material Table S4** presents the top ten highly influential co-cited journals based on publication volume and publication centrality metrics. Among these, JOURNAL OF ETHNOPHARMACOLOGY emerged as the most frequently cited journal with 835 citations, followed by JOURNAL OF AGRICULTURAL AND FOOD CHEMISTRY (454), PHYTOCHEMISTRY (409), MOLECULES (398), PHYTOTHERAPY RESEARCH (385), FOOD CHEMISTRY (383), PLANTA MEDICA (376), FITOTERAPIA (371), JOURNAL OF NATURAL PRODUCTS (330), and PHYTOMEDICINE (289). And most of which are high-impact factor journals, indicating a greater number of core journals in this field. Notably, one node on the outer edge is marked with a purple circle representing “ANTIMICROBIAL AGENTS AND CHEMOTHERAPY”, signifying its close association with other journals and attaining the highest centrality score of 0.12. The examination of microorganisms found in medicinal plants spans across various fields including medicine, agriculture, forestry science, biology, and chemistry.

#### 3.3.2 Network construction: mapping authors co-citation relationships

The network of authors frequently cited together proves to be an invaluable resource for recognizing distinguished academics and experts in various academic disciplines. As depicted in **Figure 4B**, the network comprises 949 co-cited authors connected by 4478 links. Notably, authors with significant degrees of co-citation hold pivotal positions within the realm of medicinal plant microorganisms. Out of all the authors, those who choose to remain anonymous receive an exceptional amount of citations and are positioned within the top ten in terms of co-citation frequency. **Supplementary Material Table S5** presents the top ten prolific co-cited authors based on citation count and publication centrality. Other noteworthy contributors include COWAN M.M. (134), ELOFF J.N. (91), AHMAD I. (69), RIOS J.L. (64), BAUER A.W. (64), NEWMAN D.J. (64), STROBEL G. (56), BURT S. (54), and CLSI (36). These esteemed researchers actively engage in studying medicinal plant microorganisms from diverse scientific perspectives. COWAN M.M., ELOFF J.N., AHMAD I., RIOS J.L., and BURT S researched the antibacterial properties of medicinal plants, while BAUER A.W. specializes in conducting microbiological drug sensitivity testing. NEWMAN D.J. and STROBEL G specialized in extracting natural medicinal products from microorganisms. The combined results indicate that the authors cited together have played a crucial role in promoting research and progress in the domain of microorganisms found in medicinal plants. The combined data undeniably showcase the significant impact these co-referenced authors have had on the progression of medicinal plant microorganisms research. Consequently, utilizing a multifaceted approach to author co-citation analysis allows for the delineation of the research framework related to medicinal plant microorganisms. This approach enhances scholars’ comprehensive understanding of the field’s evolution and provides essential guidance for the strategic planning of future research endeavors.

#### 3.3.3 Network construction: mapping reference co-citation relationships

The “reference” parameter within the CiteSpace analysis software was used to produce a visual depiction of the network of references pertaining to microorganisms found in medicinal plants, as depicted in **Figure 4C**. The nodes within this network correspond to distinct literature sources that have been co-cited within articles and are distinguished by their color and size. Notably, larger nodes indicate a higher frequency of citations (Hu et al., 2011). The largest node corresponds to Balouiri M, who has been cited 26 times for his significant contribution in elucidating the screening and evaluation methods of antimicrobial activity, aimed at developing novel antimicrobial drugs to combat microbial resistance (Balouiri et al., 2016). The second is Ríos J.L., who was cited 24 times for his contribution to exploring medicinal plants as potential antibacterial active pharmaceutical ingredients (APIs) and as sources of natural compounds for the development of novel anti-infective agents (Ríos and Recio, 2005). The top 10 most effective co-cited references, along with author and publication details, node, centrality, and publication year, are presented in **Supplementary Material Table S6**. Among these references, Balouir and Balouir were cited more frequently than any other authors. Other highly cited references include Newman and Cragg (2020) with 15 citations, Cos et al. (2006); Mostafa et al. (2018), and Burt (2004) each with 14 citations. Golinska et al. (2015) received 13 citations while More et al. (2008); Elisha et al. (2017), and Hardoim et al. (2015) were cited 11 times each. The majority of these research papers focus on exploring the application of naturally occurring medicinal substances obtained from medicinal plants as therapeutic antibacterial agents or the extensive utilization of endophytic bacteria residing within medicinal plants. The literature nodes associated with Rios, Cos, Burt, and more appear in a monochromatic gray hue, indicating that their research is relatively early with no recent developments. Conversely, the literature nodes corresponding to Balouiri, Newman, Mostafa, Golinska, Elisha, and Hardoim are represented in vibrant colors, indicating their more recent contributions to the field. Based on the aforementioned, a fundamental framework for research in medicinal plant microbiology has been established within this domain, while employing literature co-citation analysis to elucidate diverse perspectives on the advancement of medicinal plant microbiology and identify highly cited and representative articles.

After completing the cooperation network and co-citation analyses, a keyword analysis can be conducted to gain a deeper understanding of themes and development dynamics in the research field. This analysis will help researchers identify trends in key terms and research hotspots, providing valuable insights into emerging issues within the domain.

### 3.4 Popular research topics and trends

Keywords are essential components of scholarly articles, serving as crucial indicators that succinctly capture the central content and fundamental concept of an article (Zhang et al., 2017). In this study, CiteSpace was utilized to perform an extensive examination of the terms related to the study on microorganisms in medicinal plants, covering their co-occurrence patterns, clustering tendencies, chronological development, and emerging topics.

#### 3.4.1 Conclusion and future directions for keyword co-occurrence research

**Figure 5:**
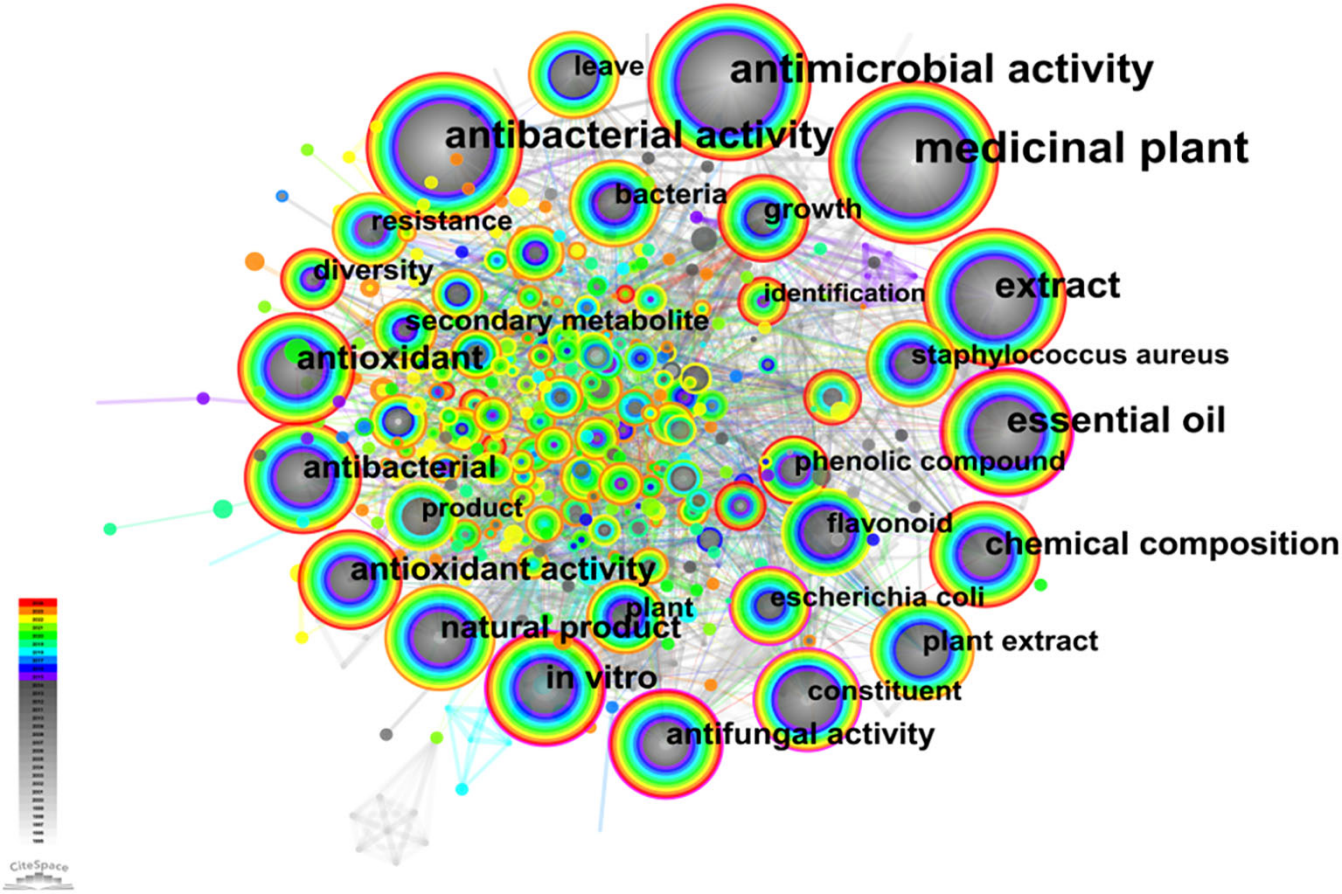
Keyword co-occurrence network on microorganisms in medicinal plant research.

The analysis of the interrelationship between keywords in the relevant literature revealed a cohesive network structure consisting of 833 nodes and 2,990 connections (**Figure 5**). Out of these, a total of ten keywords were found to have a frequency surpassing 100, as detailed in **Supplementary Material Table S7**. The keyword that appeared most frequently was “medicinal plant” (761 times), followed by “antimicrobial activity” (425 times), “antibacterial activity” (324 times), “essential oil” (232 times), “extract” (223 times), “antioxidant” (130 times), “chemical composition” (119 times), “*in vitro*” (115 times), “antioxidant activity” (113 times), and finally, “antibacterial” with 103 times. Furthermore, keywords such as “essential oil”, with a centrality value of 0.16, along with others like “*in vitro*”, “constituent”, “antifungal activity”, and “escherichia coli”, all possessing centrality values greater than 0.1 were observed within the co-occurrence network diagram. Notably, the presence of keywords such as “antimicrobial activity” and “antioxidants” on the nodes of the co-occurrence network diagram suggests their significance as major research areas within this field. Additionally, “essential oil”, “*in vitro*”, “chemical composition”, and “*in vitro*” demonstrate their frequent usage across studies conducted in this domain.

#### 3.4.2 Identification of dominant themes within keyword clusters

**Figure 6:**
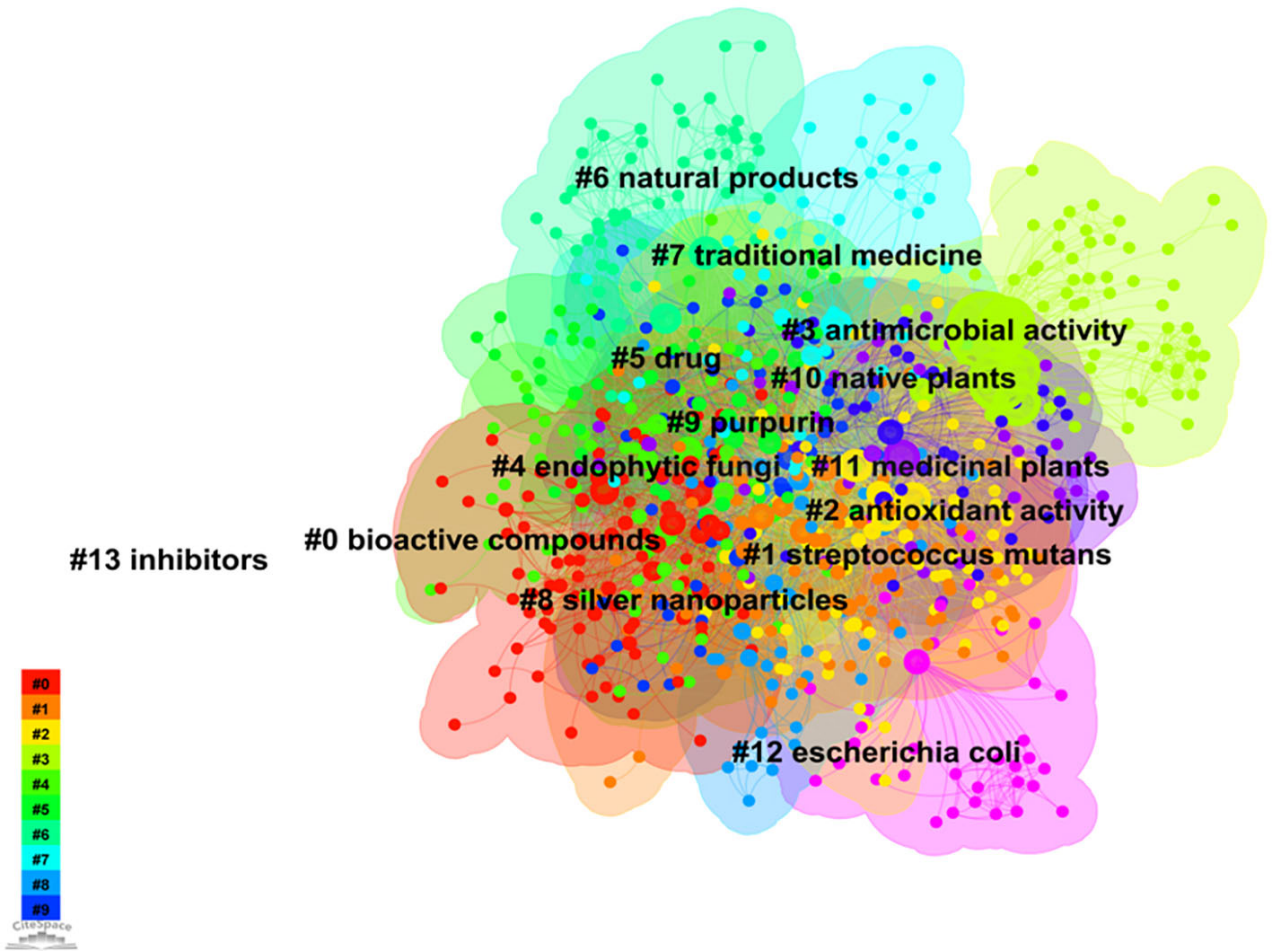
Keyword cluster network on microorganisms in medicinal plant research.

The keyword network and clustering are illustrated in **Figure 6**. CiteSpace employs two indicators, namely modularity (Q) and silhouette (S), to reconstruct the network structure and assess the clarity of clustering, thereby evaluating the visualization effectiveness. The value of Q>0.3 indicates a statistically significant clustering structure, while S>0.5 suggests a reasonably strong clustering pattern. Moreover, if S exceeds 0.7, the clustering effect can be considered highly convincing (Zhou et al., 2019). The method of keyword cluster analysis is a valuable approach for identifying the distribution and hotspots of research about microorganisms found in medicinal plants. By grouping keywords with similar characteristics, this method effectively reveals significant research directions and trends within various fields or topics. This research demonstrated excellent levels of Q (0.5586) and S (0.7706), signifying the robustness of the clustering outcomes obtained. The classification of keyword clustering comprises a total of 14 unique categories. Consequently, these findings provide reliable insights into the knowledge distribution associated with medicinal plant microorganisms.

The research on microorganisms associated with medicinal plants predominantly emphasizes the antibacterial and antioxidant properties inherent in these plants (Jaberian et al., 2013; Škrovánková et al., 2012; Vaou et al., 2021). Microorganisms contribute to the biosynthesis of specific bioactive compounds within medicinal plants and utilize the antimicrobial agents present to counteract microbial infections (Castronovo et al., 2021; Yang et al., 2016). This symbiotic interaction between medicinal plants and their associated microorganisms enhances nutrient uptake, confers resistance against pathogenic microorganisms, and promotes plant growth, ultimately improving both the yield and quality of medicinal plants (Sekar and Kandavel, 2010; Wang et al., 2022; Wu et al., 2021). For instance, plant growth-promoting rhizobacteria (PGPR) exert direct or indirect effects as growth enhancers and biocontrol agents in medicinal plants, gradually replacing the utilization of fertilizers, pesticides, and other growth regulators. The potential application of these rhizosphere microorganisms in medicinal plants is steadily expanding due to their ability to produce substantial quantities of growth-promoting chemical compounds that have an indirect impact on the overall structure of plants (Egamberdieva et al., 2015; Kumar et al., 2022; Sharma et al., 2015). Furthermore, *Streptococcus mutans*, a prevalent pathogenic bacterium associated with various diseases such as laryngitis and skin infections, exhibit close association with microorganisms found in medicinal plants. Certain active compounds present in these plants may possess inhibitory or bactericidal properties against *Streptococcus mutans*, thereby highlighting the potential of research focused on this pathogenic microorganism for medicinal applications (Vaou et al., 2021). Simultaneously, certain medicinal plants may harbor bioactive compounds capable of combating *S. mutans* infections. Consequently, investigating the correlation between medicinal plants and *S. mutans* can facilitate the development of novel antibacterial agents or extracts for treating associated infections (Murugan et al., 2013). Purpurin is a naturally occurring compound prevalent in medicinal plants such as Perilla and purple grass. It exhibits a diverse array of biological functions, including antioxidant, anti-inflammatory, and antibacterial properties. Consequently, purpurin is widely utilized in the fields of medicine and healthcare products (Singh et al., 2021). In the past few years, a collaborative effect and improved antibacterial efficacy have been observed by scientists when purpurin is combined with silver nanoparticles. In particular, the compound purpurin demonstrates its ability to transform silver ions into nanoparticles of silver through reduction, alongside displaying inherent antibacterial properties. This enhanced antibacterial efficacy is a result of the synergistic interaction between erythrosine and silver nanoparticles (Nartop, 2018). Additional research is crucial in order to discover more types of microorganisms that are linked to the growth and progress of medicinal plants, as well as to enhance our comprehension of other natural products derived from medicinal plants, microbial physiological metabolism, and plant-microbe interactions.

#### 3.4.3 Timeline visualization of keyword co-occurrence clustering analysis

**Figure 7:**
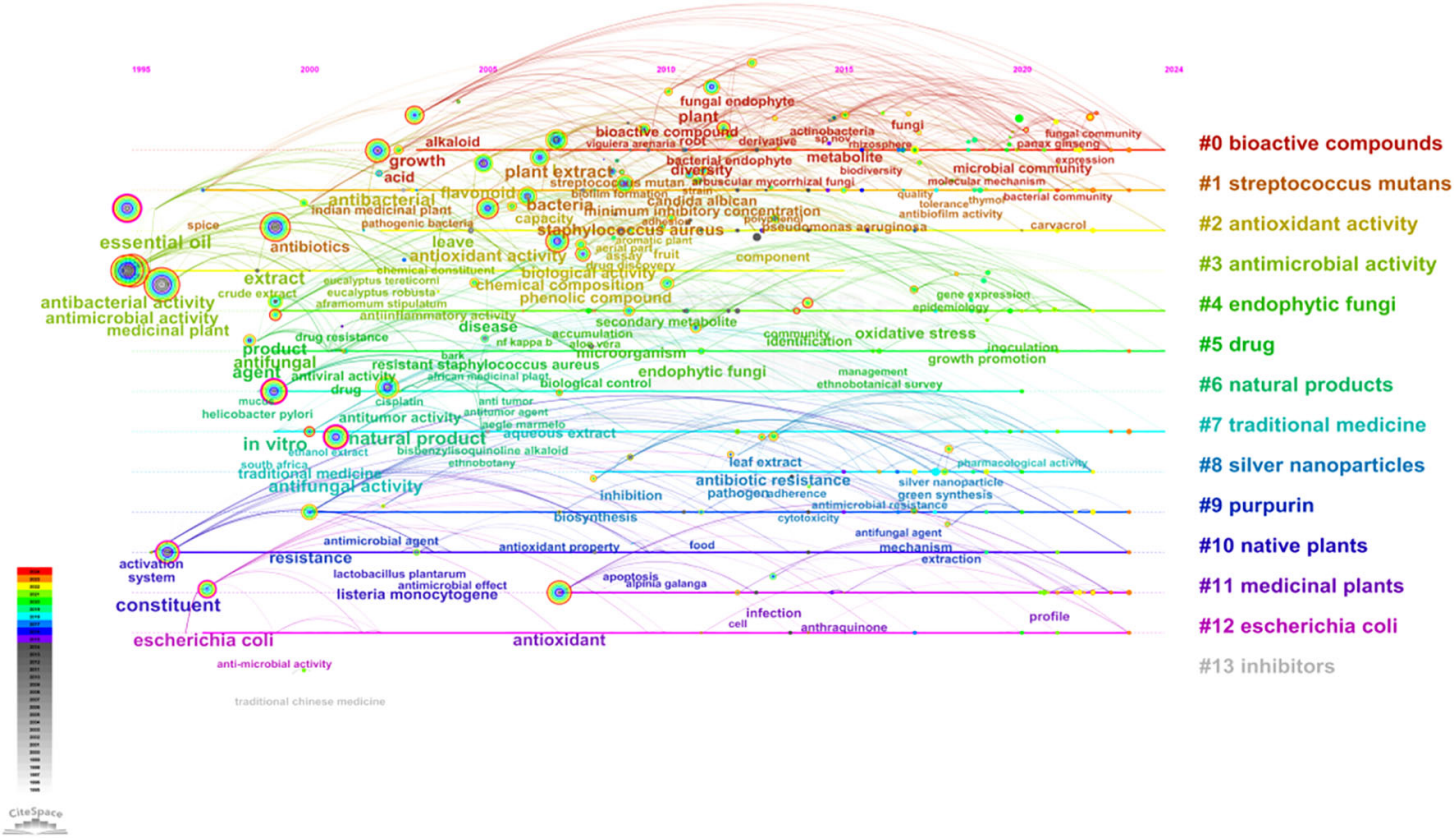
Timeline visualization of keyword cluster network on microorganisms in medicinal plant research.

The examination of the timeline for keyword clustering reveals that the research emphasis on microorganisms found in medicinal plants fluctuates across different temporal phases (**Figure 7**). In the early period of the study (1995-2002), researchers focused on Cluster#3 (antimicrobial activity), Cluster#4 (endophytic fungi), Cluster#5 (drug), Cluster#6 (natural products) and Cluster#7 (traditional medicine). Cluster#0 (bioactive compounds) became the main research hotspot around 2003, among which Cluster#1 (*streptococcus mutan*) was the most important microbial type studied. Therefore, the research of medicinal plant microorganisms mainly focused on the extraction of plant active substances and the research on the antimicrobial activity of *Streptococcus mutans* (Palombo, 2011). Subsequently, Cluster#2’s antioxidant activity has become a research hotspot. Additionally, Cluster#9 (purpurin), which is produced through the reduction reaction of anthraquinone, has gained attention. Both anthraquinone and purpurin are aromatic compounds with antibacterial, antiviral, anticancer, and other therapeutic effects. Many medicinal plants contain abundant amounts of anthraquinone compounds, and researchers are striving to explore the potential use of purpurin for related antibacterial and antioxidant activities (Sreeranjini and Siril, 2014). Cluster#8 (silver nanoparticles) has emerged as a prominent area of research, with silver nanoparticles (SNPs) finding extensive applications in the medical domain. The rapid reduction of silver ions in aqueous solutions was achieved through the utilization of medicinal plant extracts for extracellular synthesis of silver nanoparticles. The findings underscore the potential advantages of silver nanoparticles over conventional antibiotics (Chung et al., 2016; Jain and Mehata, 2017; Savithramma et al., 2011). In recent years, the incorporation of omics technologies has advanced the concurrent analysis of microbial community omics and medicinal plant metabolomics (Rai et al., 2017; Zhong et al., 2022). This advancement has enabled a comprehensive understanding of the interactions between medicinal plants and their associated microorganisms, as well as the influence of these microorganisms on the growth, metabolism, and pharmacodynamics of medicinal plants. Such an integrated approach offers significant insights for optimizing cultivation, utilization, and management practices pertaining to medicinal plants.

#### 3.4.4 A study of historical and current trends in keyword burst analysis

**Figure 8:**
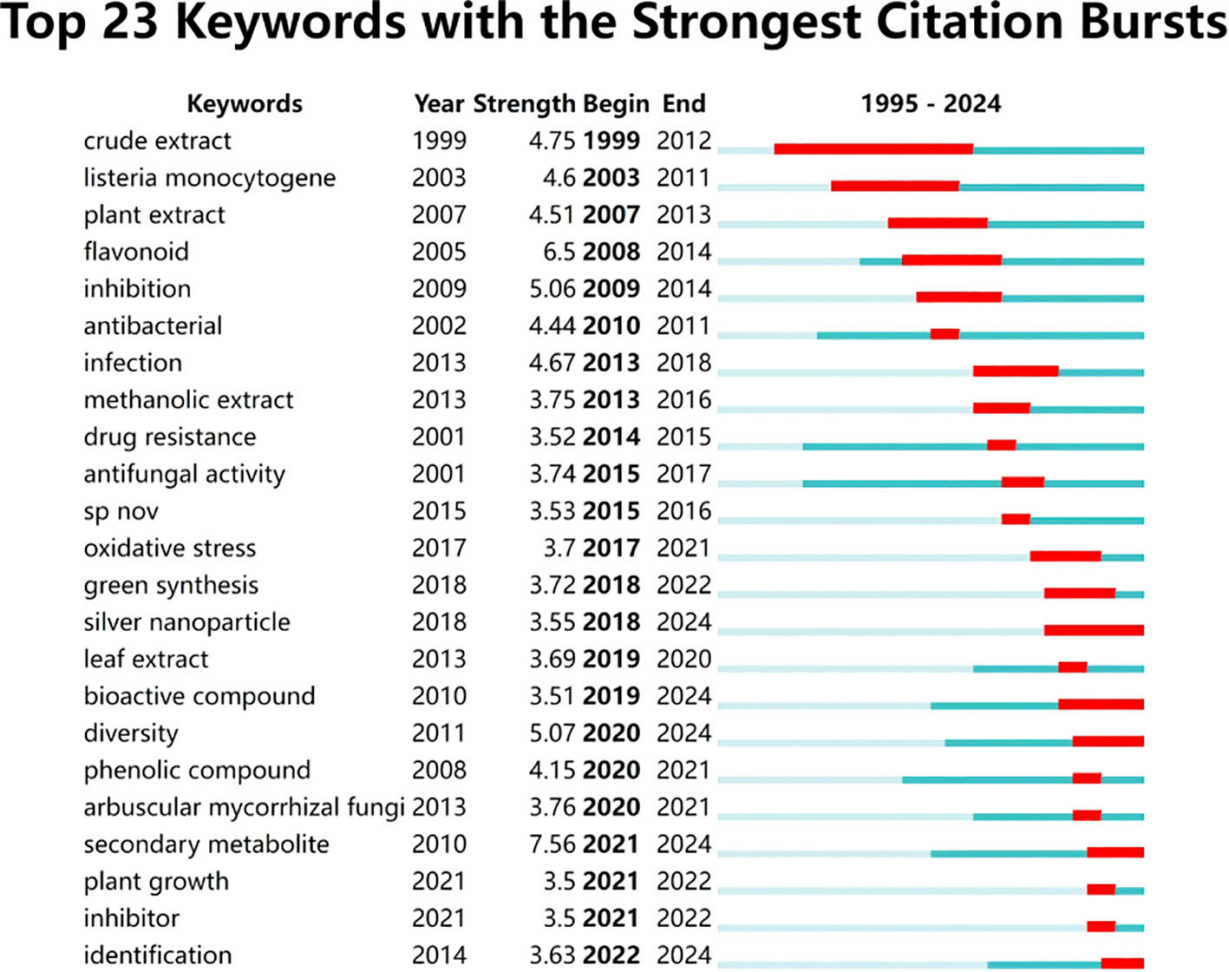
Top 23 keywords with the strongest citation bursts on microorganisms in medicinal plant research.

Keyword bursts denote a notable increase in the search or utilization frequency of certain keywords within a particular time frame, typically reflecting changes in hot topics or trends. The visualization results are depicted in **Figure 8**. Among the top 10 citation keywords exhibiting the most pronounced bursts, four keywords stand out: “extract” (1999-2016), “listeria monocytogene” (2003-2011), “flavonoid” (2008-2014), and “antibacterial” (2010-2018). Notably, the term with the longest burst is “crude extract”, which has maintained its popularity for a remarkable span of 13 years. Furthermore, recent captivating keywords include “secondary metabolite” and “identification”, both of which continue to experience ongoing bursts as of 2024. Over time, medicinal plant microorganisms have evolved from being primarily associated with antibacterial activity derived from natural products to encompassing intricate interactions between microbiota and secondary metabolites (Mishra et al., 2022). This advancement underscores the significance of microbial analysis technology and omics technology as indispensable tools for investigating plant-microbe interactions while simultaneously guiding future research directions and topic selection about medicinal plant microorganisms.

## 4 Conclusions and outlook

**Figure 9:**
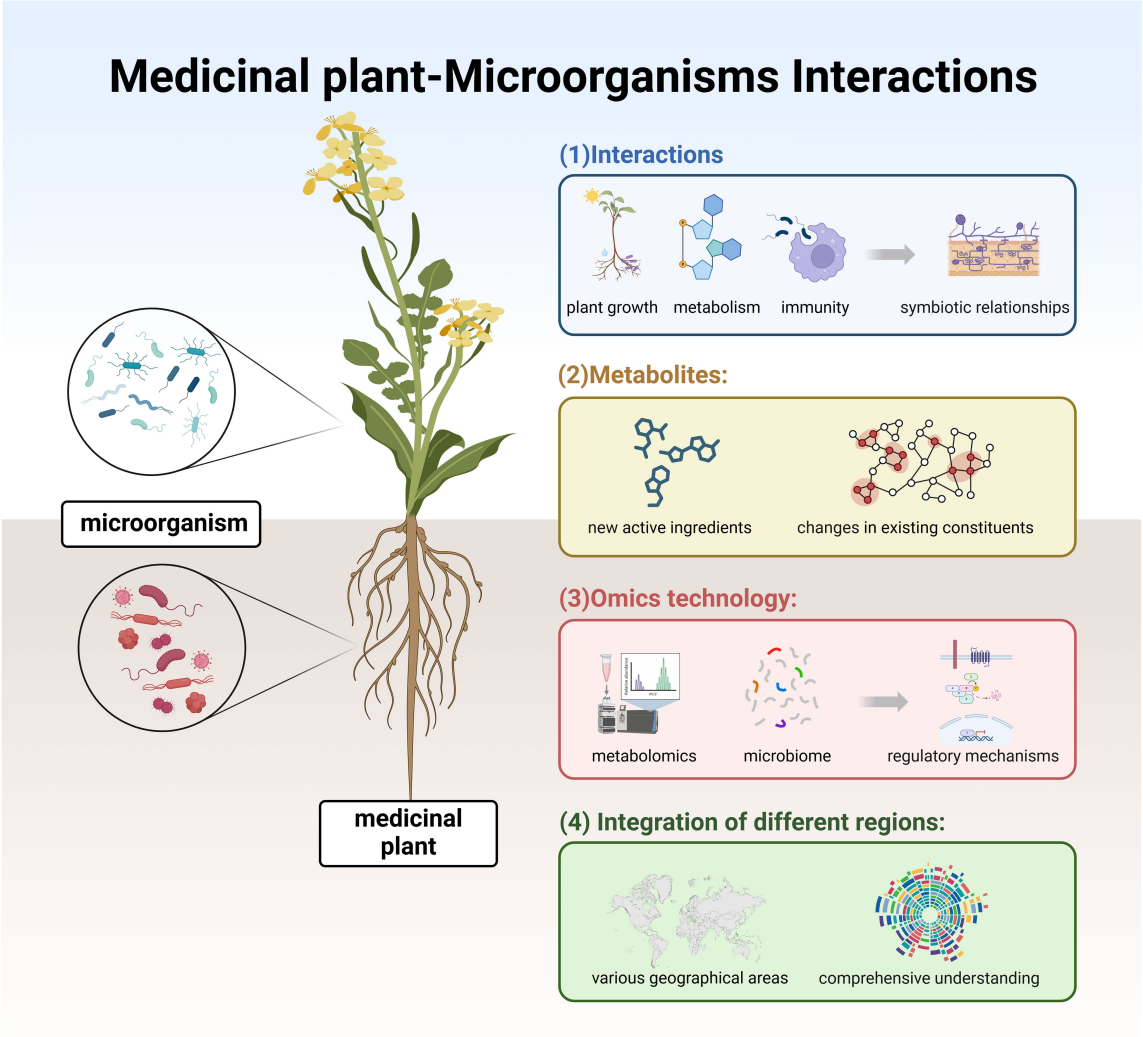
Medicinal plant - microorganisms interactions.

The paper presents the utilization of CiteSpace, a software for visualizing scientific information, utilizing the scientific knowledge atlas research method to visually analyze 1269 related literature on medicinal plant microorganisms from the WoS core collection database. The quantitative statistical analysis is conducted using pioneering information systems and theoretical research methods in this field, effectively presenting a comprehensive overview of medicinal plant microorganisms including country distribution, institutions, authors, co-cited journals, co-cited authors, co-cited literature, research hotspots, and frontier issues. In terms of international collaboration network, India exhibits the largest number of publications but lacks international collaboration; whereas China demonstrates both the highest publication count and close international cooperation. An examination of the publishing institution network identifies three primary core research networks: King Saud University, Islamic Azad University, and the University of Sao Paulo. Notably, the top five authors with the most published papers are all Brazilian researchers, highlighting Brazil’s substantial contribution to contemporary studies on medicinal plant microorganisms. By analyzing research hotspots and core evolution paths along with relevant articles and reviews, future in-depth investigations are proposed that provide a novel theoretical perspective for further exploration into medicinal plant microorganisms (**Figure 9**).

(1) Interactions between plants and microorganisms: the complex interactions between medicinal plants and microorganisms involve multiple dimensions, including plant growth, metabolism, immunity, and more. Notably, specific microorganisms form symbiotic relationships with medicinal plants. For example, rhizosphere nitrogen-fixing bacteria participate in mutualistic nitrogen-fixation processes by supplying essential nitrogen to plants, while mycorrhizal fungi establish symbiotic associations with plant roots to improve nutrient absorption. Further investigation into the mechanisms underlying these plant-microorganism interactions is warranted.

(2) Synthesis and extraction of metabolites: the presence of microorganisms can influence some medicinal plants’ secondary metabolite production, potentially leading to the generation of new active ingredients or changes in existing constituents. With advancements in technology, more efficient, green, and sustainable extraction methods for natural products will emerge, such as ultrasonic extraction, microwave-assisted extraction, and ionic liquid extraction, improving extraction efficiency and product purity.

(3) Joint research utilizing omics technology: by integrating metabolomics analysis of medicinal plants and profiling microbial communities, a deeper understanding of the isolation of organic compounds can be achieved. This approach can elucidate the involvement and regulatory mechanisms of microorganisms in the production of these compounds, providing valuable insights into the conditions under which medicinal plants thrive and how they produce pharmacologically active compounds, thus aiding in the development and application of herbal remedies.

(4) Joint analysis of microorganisms associated with medicinal plants across different regions: different regions possess unique medicinal plant resources, and current research primarily focuses on the interactions among medicinal plants, microorganisms, and natural products within individual countries. Due to geographical and climatic differences, distinct medicinal flora exist, but the types of microbial communities interacting with them generally remain consistent. Therefore, future research efforts should emphasize a holistic examination across various geographical areas to gain a more comprehensive understanding of the relationships and mechanisms between identical medicinal plant species and microorganisms.
