# Supplementary material for: Bibliometric analysis of research trends and advancements in medicinal plant microbiome
Source: Front Plant Sci. 2024 Nov 20;15:1495198. doi: 10.3389/fpls.2024.1495198 (PMC11614653; doi:10.3389/fpls.2024.1495198)
Supplement: Supplementary file 1 [file Table1.docx]

**Supplementary material**

**Table S1.** Top 10 countries in terms of publications on microorganisms in medicinal plant research.

**Table S2.** Top 10 institutions in terms of publications on microorganisms in medicinal plant research.

**Table S3.** Top 10 most productive authors of microorganisms in medicinal plant research.

**Table S4.** Top 10 most cited journals of microorganisms in medicinal plant research.

**Table S5.** Top 10 most cited authors of microorganisms in medicinal plant research.

**Table S6.** Top 10 most cited references of microorganisms in medicinal plant research.

**Table S7.** Top 10 keyword co-occurrence of microorganisms in medicinal plant research.

**Table S1.** Top 10 countries in terms of publications on microorganisms in medicinal plant research.

| No. | Countries | Count | Centrality | Year |
| --- | --- | --- | --- | --- |
| 1 | India | 230 | 0 | 1998 |
| 2 | Brazil | 151 | 0.07 | 1997 |
| 3 | Peoples R China | 111 | 0.26 | 2000 |
| 4 | Turkey | 86 | 0 | 1999 |
| 5 | Saudi Arabia | 68 | 0.09 | 2009 |
| 6 | Pakistan | 67 | 0 | 2004 |
| 7 | Iran | 61 | 0.03 | 2003 |
| 8 | South Africa | 59 | 0.13 | 1999 |
| 9 | Egypt | 50 | 0 | 1997 |
| 10 | USA | 42 | 0.42 | 2001 |

**Table S2.** Top 10 institutions in terms of publications on microorganisms in medicinal plant research.

| No. | Institution | Count | Centrality | Year |
| --- | --- | --- | --- | --- |
| 1 | King Saud Univ | 20 | 0.01 | 2014 |
| 2 | Islamic Azad Univ | 17 | 0 | 2008 |
| 3 | Univ Sao Paulo | 13 | 0 | 2000 |
| 4 | Univ Mauritius | 9 | 0 | 2005 |
| 5 | Ankara Univ | 9 | 0 | 2004 |
| 6 | Univ Fed Paraiba | 9 | 0 | 2003 |
| 7 | Univ Franca | 9 | 0 | 2009 |
| 8 | Ataturk Univ | 7 | 0 | 2003 |
| 9 | Bulgarian Acad Sci | 7 | 0 | 2011 |
| 10 | Univ Pretoria | 7 | 0 | 2008 |

**Table S3.** Top 10 most productive authors of microorganisms in medicinal plant research.

| No. | Authors | Count | Centrality | Year | From |
| --- | --- | --- | --- | --- | --- |
| 1 | Furtado, Niege A J C | 5 | 0 | 2009 | Brazil |
| 2 | Martins, Carlos H G | 5 | 0 | 2009 | Brazil |
| 3 | Veneziani, Rodrigo C S | 5 | 0 | 2009 | Brazil |
| 4 | Ambrosio, Sergio R | 5 | 0 | 2009 | Brazil |
| 5 | Heleno, Vladimir C G | 5 | 0 | 2009 | Brazil |
| 6 | Mahomoodally, Mohamad Fawzi | 4 | 0 | 2021 | Vietnam |
| 7 | Al-dhabi, Naif Abdullah | 4 | 0 | 2012 | Saudi Arabia |
| 8 | Zengin, Gokhan | 4 | 0 | 2021 | Turkey |
| 9 | Abdallah, Emad M | 4 | 0 | 2023 | Saudi Arabia |
| 10 | Adeleke, Rasheed | 3 | 0 | 2021 | South Africa |

**Table S4.** Top 10 most cited journals of microorganisms in medicinal plant research.

| No. | Journals | Count | Centrality | Year | Impact Factor |
| --- | --- | --- | --- | --- | --- |
| 1 | J. Ethnopharmacol. | 835 | 0.04 | 1995 | 5.4 |
| 2 | J. Agric. Food Chem. | 454 | 0.02 | 1997 | 6.1 |
| 3 | Phytochemistry | 409 | 0.02 | 1999 | 3.8 |
| 4 | Molecules | 398 | 0.02 | 2009 | 4.6 |
| 5 | Phytother. Res. | 385 | 0.02 | 1995 | 7.2 |
| 6 | Food Chem. | 383 | 0.02 | 2007 | 8.8 |
| 7 | Planta Med. | 376 | 0.01 | 1995 | 2.7 |
| 8 | Fitoterapia | 371 | 0.04 | 1995 | 3.4 |
| 9 | J. Nat. Prod. | 330 | 0.07 | 1999 | 5.1 |
| 10 | Phytomedicine | 289 | 0.04 | 2003 | 7.9 |

**Table S5.** Top 10 most cited authors of microorganisms in medicinal plant research.

| No. | Authors | Count | Centrality | Year |
| --- | --- | --- | --- | --- |
| 1 | [ANONYMOUS] | 400 | 0.11 | 1998 |
| 2 | COWAN MM | 134 | 0.16 | 2005 |
| 3 | ELOFF JN | 91 | 0.17 | 2000 |
| 4 | AHMAD I | 69 | 0.14 | 2002 |
| 5 | RÍOS JL | 64 | 0.09 | 2007 |
| 6 | BAUER AW | 64 | 0.17 | 1995 |
| 7 | NEWMAN DJ | 64 | 0.06 | 2010 |
| 8 | STROBEL G | 56 | 0.06 | 2011 |
| 9 | BURT S | 54 | 0.06 | 2008 |
| 10 | CLSI | 36 | 0.02 | 2010 |

**Table S6.** Top 10 most cited references of microorganisms in medicinal plant research.

| No. | Authors | Count | Centrality | Year |
| --- | --- | --- | --- | --- |
| 1 | Balouiri M, 2016, J PHARM ANAL, V6, P71, DOI 10.1016/j.jpha.2015.11.005 | 26 | 0.09 | 2016 |
| 2 | Ríos JL, 2005, J ETHNOPHARMACOL, V100, P80, DOI 10.1016/j.jep.2005.04.025 | 24 | 0.28 | 2005 |
| 3 | Newman DJ, 2020, J NAT PROD, V83, P770, DOI 10.1021/acs.jnatprod.9b01285 | 15 | 0.02 | 2020 |
| 4 | Cos P, 2006, J ETHNOPHARMACOL, V106, P290, DOI 10.1016/j.jep.2006.04.003 | 14 | 0.07 | 2006 |
| 5 | Mostafa AA, 2018, SAUDI J BIOL SCI, V25, P361, DOI 10.1016/j.sjbs.2017.02.004 | 14 | 0.1 | 2018 |
| 6 | Burt S, 2004, INT J FOOD MICROBIOL, V94, P223, DOI 10.1016/j.ijfoodmicro.2004.03.022 | 14 | 0.01 | 2004 |
| 7 | Golinska P, 2015, ANTON LEEUW INT J G, V108, P267, DOI 10.1007/s10482-015-0502-7 | 13 | 0.39 | 2015 |
| 8 | More G, 2008, J ETHNOPHARMACOL, V119, P473, DOI 10.1016/j.jep.2008.07.001 | 11 | 0.06 | 2008 |
| 9 | Elisha IL, 2017, BMC COMPLEM ALTERN M, V17, P0, DOI 10.1186/s12906-017-1645-z | 11 | 0.02 | 2017 |
| 10 | Hardoim PR, 2015, MICROBIOL MOL BIOL R, V79, P293, DOI 10.1128/MMBR.00050-14 | 11 | 0.03 | 2015 |

**Table S7.** Top 10 keyword co-occurrence of microorganisms in medicinal plant research.

| No. | Keywords | Count | Centrality | Year |
| --- | --- | --- | --- | --- |
| 1 | medicinal plant | 761 | 0.05 | 1996 |
| 2 | antimicrobial activity | 425 | 0.07 | 1995 |
| 3 | antibacterial activity | 324 | 0.09 | 1995 |
| 4 | essential oil | 232 | 0.16 | 1996 |
| 5 | extract | 223 | 0.09 | 1999 |
| 6 | antioxidant | 130 | 0.09 | 2007 |
| 7 | chemical composition | 119 | 0.04 | 2007 |
| 8 | in vitro | 115 | 0.17 | 1999 |
| 9 | antioxidant activity | 113 | 0.06 | 2005 |
| 10 | antibacterial | 103 | 0.09 | 2002 |
